# Supplementary material for: MetaRibo-Seq measures translation in microbiomes
Source: Nat Commun. 2020 Jun 29;11:3268. doi: 10.1038/s41467-020-17081-z (PMC7324362; doi:10.1038/s41467-020-17081-z)
Supplement: Supplementary file 10 — Supplementary Data 7 [file 41467_2020_17081_MOESM10_ESM.zip › File2/Confidence_High_Taxonomy/194425_out.krona.html]

Javascript must be enabled to view this page.

members
magnitude
magnitudeUnassigned
count
unassigned
taxon
rank

194425\_out

3

2
3
superkingdom

phylum
3
1239

909932
1
class

order
1843489
1

1
31977
family

39948
1
genus

species
1

SRS1055069\_contig\_number\_10571
218538

class
186801
2

2
186802
order

family
2
541000

1263
2
genus

2

SRS142923\_contig\_number\_19002SRS148424\_contig\_number\_contig-100\_5.168596
165186
species
